# Effects of micro-osteoperforations performed with Propel system on tooth movement, pain/quality of life, anchorage loss, and root resorption: a systematic review and meta-analysis

**DOI:** 10.1186/s40510-020-00326-4

**Published:** 2020-07-27

**Authors:** Cibelle Cristina Oliveira dos Santos, Paulo Mecenas, Mônica Lidia Santos de Castro Aragón, David Normando

**Affiliations:** 1 Post-graduation program of dentistry, Federal University of Pará (UFPA), Rua Augusto Correa 01, Belém, Pará 66075-110 Brazil; 2 Department of Orthodontics, Dental School, Federal University of Pará (UFPA), Belém, Pará Brazil

**Keywords:** Canine retraction, Tooth movement techniques, Micro-osteoperforation, MOPs, Orthodontics

## Abstract

**Background:**

The aim of this systematic review was to evaluate the effect of micro-osteoperforations (MOPs) performed with Propel and other mini-screws on the rate of tooth movement, pain/discomfort, periodontal health, anchorage loss, and root resorption in patients undergoing orthodontic retraction compared to a control group.

**Materials and methods:**

PubMed, Cochrane, Web of Science, LILACS, Google Scholar, Scopus, and OpenGrey were searched without restriction. A manual search was also carried out. Only randomized clinical trials (RCT) were included. The risk of bias (RoB) was assessed using RoB 2.0 and the certainty of evidence through the GRADE tool.

**Results:**

Among the twelve RCTs reviewed, five used the Propel system. Overall, the RoB was classified as low (4), moderate (5), and high (3). Two RCTs with moderate and one with a low RoB using the Propel system reported mild increases on rate of tooth movement associated with MOPs. One RCT with a moderate and another with high RoB did not find a significant effect of Propel on orthodontic movement. Regarding tooth movement, a subgroup meta-analysis found no differences between control and Propel movement (95% CI = − 0.01 to 0.75) or other mini-screws (− 0.02 to 0.31) related to rate of tooth movement per month. There was no effect of MOPs on root resorption, periodontal health, anchorage loss, and a mild effect on pain and oral health related to quality of life regardless of mini-screw type. The level of certainty was graded as low for the rate of tooth movement and pain/discomfort, as moderate for anchorage loss, and high for root resorption.

**Conclusion:**

A low certainty of evidence supports that MOPs performed with Propel seem to have no significant effect on the rate of tooth movement. Moreover, this intervention does not seem to cause an increase in root resorption, periodontal heath, pain/discomfort, or anchorage loss. Thus, the Propel system does not appear to produce different results from those observed for other mini-screws.

## Introduction

With the aim of reducing orthodontic treatment time and side effects such as root resorption [1], pain [2], and impact on quality of life [3], several techniques used to accelerate tooth movement have excelled in orthodontics [4–6]. Some approaches cause cortical bone injuries and consequently increase the expression of inflammatory mediators, precursors of bone remodeling associated with orthodontic movement [7–9], such as corticotomies and micro-osteoperforations (MOPs).

Corticotomies are performed to reduce biological resistance to tooth movement and orthodontic treatment time [10]. A recent meta-analysis showed a reduction in total treatment time associated with corticotomies by approximately 2.8 months and a decline in rate of tooth movement after the first month of the procedure [7].

Less invasive than corticotomies, MOPs are performed using mini-screws without surgical flaps and with acceptable patient discomfort [4]. Some types of mini-screws have been used to produce MOPs. Propel (Propel Orthodontics, Ossining, NY) is a 1.4-mm surgical stainless steel mini-screw implant attached to a driver to create MOPs, which according to the company has a design to accelerate the rate of tooth movement [11]. Although, some studies have used this system to perform MOPs and reported a 2–3-fold increase in rate of movement [12, 13]. Nevertheless, MOPs using conventional mini-screws following a methodology similar to the Propel system reported no impact on tooth movement [14, 15].

Recent systematic reviews evaluated the influence of MOPs on the rate of tooth movement. However, to date, no comparative evaluation has been conducted to examine whether the Propel system could produce a different outcome than those observed when MOPs are performed with conventional mini-screws. These previous reviews have shown controversial findings and they have mixed Propel studies with others in the same meta-analysis, which is not appropriate considering the methodological heterogeneity [16, 17]. One review pointed out an increase of 0.45 mm per month on the rate of canine retraction when compared to a no-intervention group [16]; however, four studies presented in the literature were not included [15, 18–20] in its analysis. In contrast, two other reviews stated that MOPs do not accelerate tooth movement [17, 21]: one [21] with only two studies [14, 15] and another [17] with three studies [12, 14, 18]. Therefore, the aim of this systematic review was to evaluate the effects of MOPs on the rate of tooth movement, considering the use of the Propel system and possible side effects inherent to the procedure.

## Material and methods

### Protocol and registration

This study was registered at PROSPERO database (https://www.crd.york.ac.uk/prospero/display_record.php?RecordID = 113050) under registration code CRD42018113050 and was performed in accordance to the PRISMA [22] recommendations (Preferred Reporting Items for Systematic Review and Meta-Analysis).

### Eligibility criteria

The following selection criteria were adopted in accordance with the PICOS format:

- Population (P): orthodontic patients with permanent dentition who underwent premolar extraction, and canine retraction or total anterior retraction
- Intervention (I): MOPs during canine retraction or total anterior retraction
- Comparison (C): canine retraction or total anterior retraction without MOPs or other interventions
- Outcome (O): the primary outcome was the rate of tooth movement measured by the amount of canine retraction or total anterior retraction. Secondary outcomes were as follows: quality of life, impact on patient’s daily routine, and adverse effects such as pain/discomfort, root resorption, periodontal health, and anchorage loss.
- Study design (S): randomized clinical trials (RCTs)

Exclusion criteria included studies which evaluated other types of tooth movement acceleration therapy. Opinion articles, animal studies, laboratory studies, case reports, case-series, and literature reviews were also excluded.

### Information sources

The following databases were searched: PubMed, Cochrane, Web of Science, LILACS, Google Scholar, Scopus, and OpenGrey. The searches were performed until May 2020. A hand search was also carried out of the reference lists of the selected articles. No language or date publication restriction has been applied.

### Search strategy and study selection

Two independently reviewers (CS and PM) searched the databases. In cases of unresolved disagreements, a third author (DN) was consulted. The search strategy was created from a combination of MeSH, Entry terms, and Keywords related to the PICO strategy using Boolean operators (Appendix).

The reference manager software was used to save the citations (Mendeley Ltd., 2019, Elsevier). After the duplicates were removed, article titles and abstracts were read to select studies. Those relevant were analyzed by reading the full text and a final selection was done by two researchers (CS and PM). If discrepancies were unsolved, a third researcher (DN) was consulted.

### Data collection process

Two authors (CS and DN) performed the data extraction independently. The following items were considered for data extraction: author, year, location, type of study; sample size, male/female, age; type of malocclusion, retraction model, MOP protocol, utilization of Propel system, follow-up period and losses, rate of tooth movement, outcomes evaluated (rate of tooth movement, periodontal health, pain/discomfort, root resorption, anchorage loss), outcome measurements, and conclusions.

### Summary measures

The rate of tooth movement was considered the main outcome. Data was also collected on the system used to perforate, loss of anchorage, periodontal health, root resorption, pain and discomfort, and implications on the quality of life.

### Risk of bias in individual studies

Cochrane risk of bias tool (RoB 2.0) was used [23]. This tool assesses possible bias in five domains: bias arising from the randomization process, bias due to deviations from the intended interventions, bias due to missing outcome data, bias in the measurement of the outcome, and bias in the selection of the reported results. The bias was judged for each domain and to an overall evaluation of low, some concerns or high. Each risk of bias (RoB) analysis was done by two reviewers (CS and PM) and in case of discordance a third reviewer (DN) was consulted.

### Level of evidence

The certainty of scientific evidence of outcome was evaluated using the *Grading of Recommendations Assessment*, *Development*, *and Evaluation* (GRADE) [24]. The outcomes assessed were rate of tooth movement, pain and discomfort, anchorage loss, and root resorption. The articles were evaluated considering their design, RoB, consistency, directness, and precision.

## Results

### Study selection

**Fig. 1:**
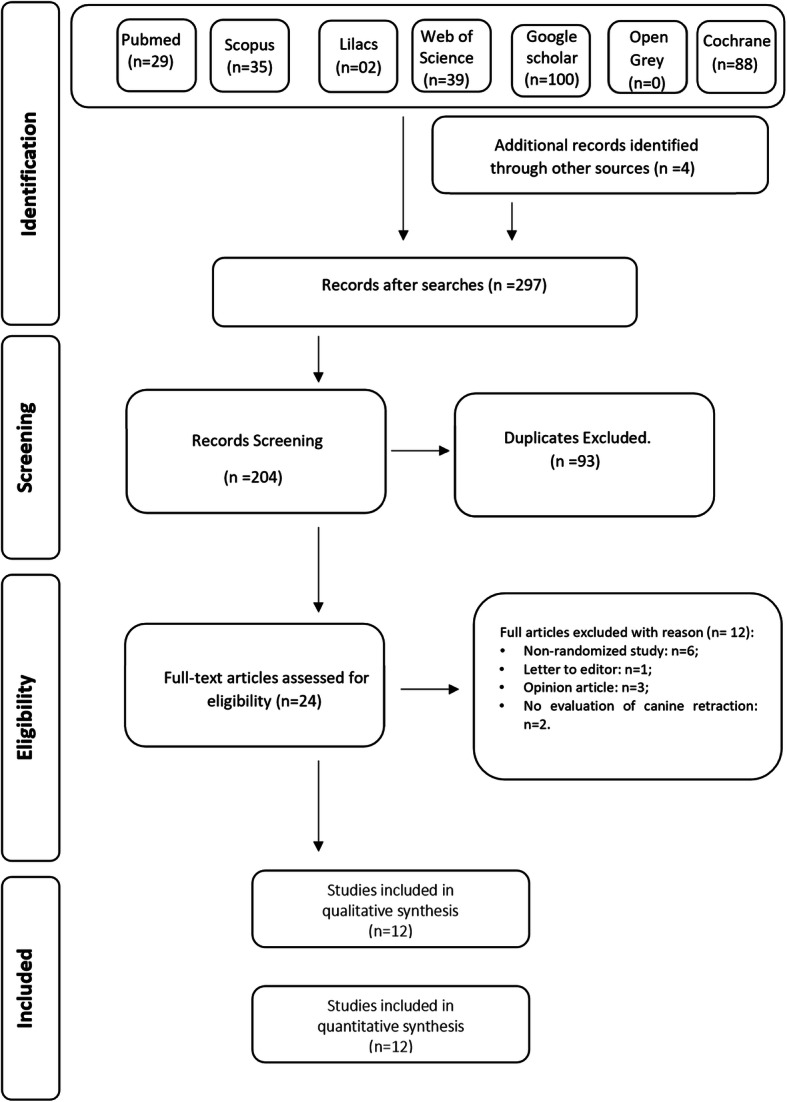
Flowchart of studies identification

The searches yielded 297 references: PubMed (*n* = 29), Scopus (*n* = 35), Cochrane (*n* = 88) Web of Science (*n* = 39), LILACS (*n* = 2), Google Scholar (*n* = 100), OpenGrey (*n* = 0), and four additional references were identified through the manual search. After removing duplicates, the title and abstract of 204 articles were read and at the end articles were evaluated by full text. Twelve were excluded after the full text was read. The reasons for exclusion are described in Table 1. Twelve RCTs were included for qualitative and quantitative synthesis. The process of identification, selection, and exclusion of studies are presented in a flowchart (Fig. 1).

**Table 1:** List of excluded studies with reason

Reference	Reason for exclusion
Abdelhameed et al. (2018)	Non-randomized study
Alikhani et al. (2014)	Opinion article
Alikhani et al. (2015)	Opinion article
Bajath et al. (2019)	Non-randomized study
Bansal et al. (2019)	Did not evaluate canine retraction
Charavet et al. (2019)	Non-randomized study
Elkalza et al. (2018)	Non-randomized study
Gulduren et al. (2020)	Did not evaluate canine retraction
Laraway et al. (2018)	Non-randomized study
Mahamoudi et al. (2016)	Non-randomized study
Prasad et al. (2014)	Letter to editor
Sangsuwon et al. (2018)	Opinion article

### Study characteristics

Five studies [12, 13, 19, 20, 25] using the Excellerator instrument (Propel Orthodontics—Ossining, NY, USA) evaluated the effects of MOPs on the rate of tooth movement in orthodontic retraction compared to a control group without MOPs; also, seven RCTs [14, 15, 18, 26–29] using other mini-screws performed the same evaluation.

Among eight studies which analyzed the pain associated with the interventions [12–15, 20, 25, 28, 29], one evaluated the impact on the daily routine of patients [14] and three studies evaluated the presence of root resorption [14, 15, 28]. None used the Propel system. One Propel study assessed the implications on quality of life [19]. Five studies, including one Propel [13], evaluated the occurrence of anchorage loss [13–15, 18, 28]. Two studies without Propel assessed the periodontal health associated to MOPs [14, 28].

Eight RCTs [12, 14, 15, 20, 26–29] used the split-mouth model and four [13, 18, 19, 25] utilized the two-arm parallel groups. The sample sizes ranged from 8 [28] to 60 [25] patients. All trials reported participants with complete permanent dentition and the mean age ranged from 14 [18] to 32 [20] years.

Regarding the type of brackets, one study used self-ligating Roth system 0.022″ [19], eight studies used MBT 0.022″ [12–14, 18, 25, 26, 28, 29], two used Roth prescription 0.022″ [15, 27], and one study used Standard edgewise 0.022″ [20]. Concerning the canine retraction, eight RCTs [12, 14, 15, 18–20, 28, 29] used mini-screws as anchorage devices and NiTi springs with strength levels between 100 and 150 g, one used mini-screws and chain elastic for retraction with forces between 140 and 200 g [26], two used conventional anchorage and NiTi springs [13, 27], and one study used transpalatal bars and tie back mechanics [25].

Regarding the number of perforations, all five Propel studies [12, 13, 19, 20, 25] and six other investigations [14, 15, 18, 26, 28, 29] were performed with three and one study with two perforations [27]. The depth of perforation ranged from 1 mm [29] to 8 mm [15]; also some studies reported 2 to 3 mm [12, 13, 25–27], 3 to 4 mm [14], 5 mm [18, 20], and 5 to 7 mm [19, 28] of depth. Thus, depth perforation among studies examining the Propel system ranged from 2-3 mm [12, 13, 25] to 7 mm [19]. Three studies, including two Propels, performed MOPs monthly throughout the retraction period [18, 19, 25], while the others [12–15, 20, 26–29] made the perforations only at the beginning of retraction. The MOPs were performed between the canine and the second premolar, vertically equidistant from each other and ranged from 2 mm [22], 3 mm [14], and 4 mm [19]; also, the first perforation was performed from 5 mm [23, 29] to 6 mm [14] to the gingival margin.

The rate of tooth movement was measured by clinical inspections and digital caliper measurements [15, 25], measurements on plaster models [12, 29] or scanned models [13–15, 18, 25, 28], computed tomography [15, 28], and panoramic radiography associated to scanned models [20].

Considering the follow-up periods, two studies [12, 23] evaluated the first month after MOPs were performed; two [18, 20] evaluated for 2 months, three [14, 26, 28] for 3 months, two [13, 15] for 4 months, and two [19, 25] until space closure. Regarding the Propel studies, one evaluated the tooth movement for 1 month [12], one for 2 months [20], one for 4 months [13], and two for the entire time of space closure in anterior retraction [19, 25].

Ten studies measured the rate of tooth movement during the canine retraction [12–15, 18, 20, 26–29] and two studies during the total space closure of the extraction, corresponding to orthodontic movement of total anterior retraction [19, 25].

### Risk of bias within studies

**Fig. 2:**
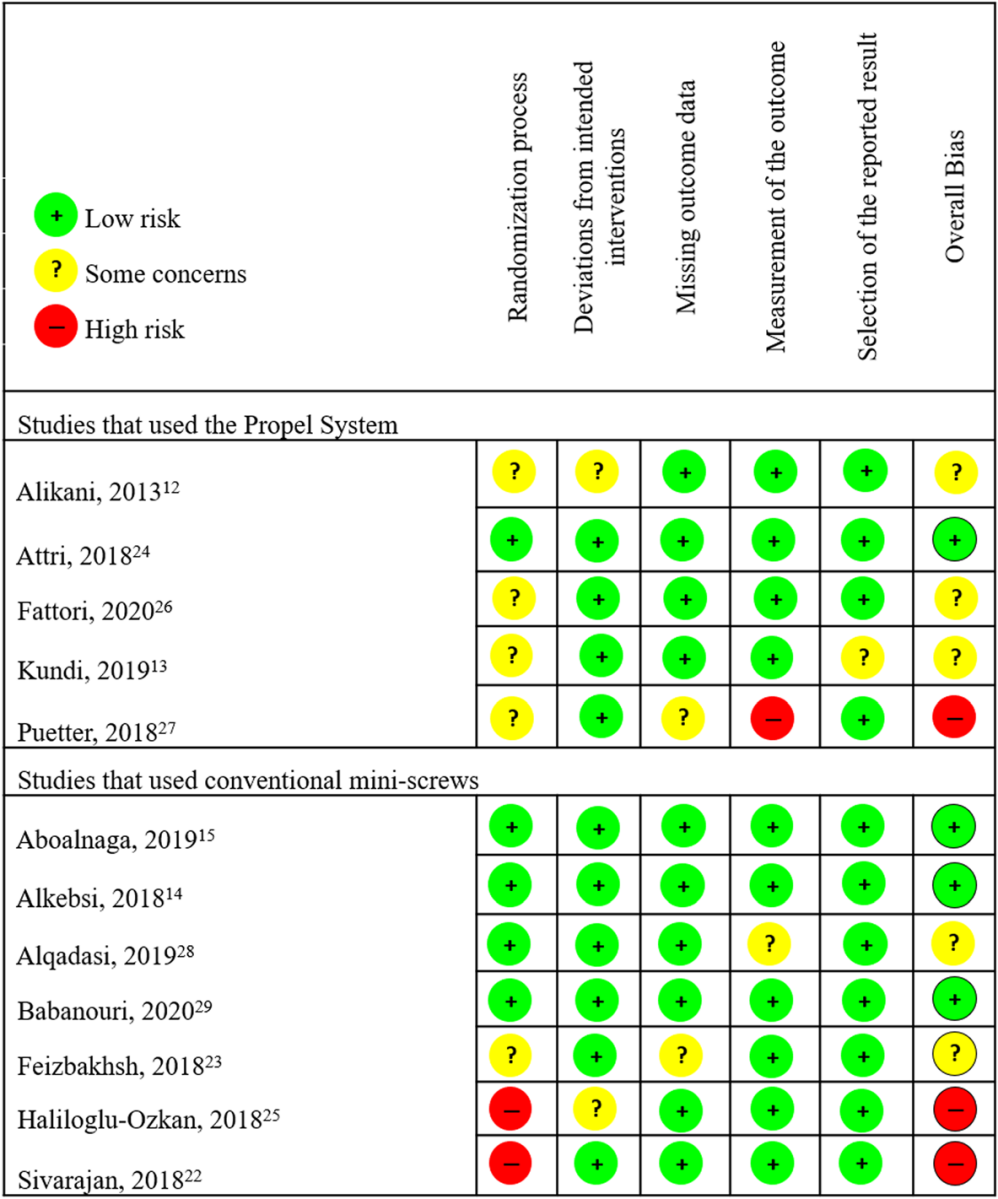
Evaluation of included studies based on RoB assessment tool RoB 2.0

Overall, four RCTs were classified with a low RoB, including two that used Propel, [14, 15, 25, 29], five were classified as some concerns, including two which used Propel [12, 13, 19, 27, 28], and three showed a high RoB, including one that used Propel [18, 20, 26]. The main causes of RoB were due to confounding factors in the randomization process [12, 13, 18–20, 26, 27], deviations from intended interventions [12, 18], missing outcome data [20, 27], measurement of the outcome [20, 28], and selection of the reported result [13]. No article was able to maintain double-blind analysis due to the nature of the study. Figure 2 shows the RoB evaluations of the included studies.

### Results of individual studies

Among five studies that used the Propel system, three [12, 13, 25] reported an increase in the rate of tooth movement, one with a low RoB [25], and two with a moderate RoB [12, 13]. Another study [20] with a high and another [19] with a moderate RoB reported no difference between groups. Seven studies used conventional mini-screws to perform the MOPs. Four of these [18, 26, 27, 29] reported an increase in the rate of orthodontic movement, while three [14, 15, 28] found no difference between groups, two with a low RoB [14, 15], and one with a moderate RoB [28]. The summary of the included studies characteristics is described in Table 2.

**Table 2:** Summary of the data from the included studies

Author, year, and study design	Sample size (*n*), M/F (*n*), and mean age ± SD	Malocclusion	Bracket system, Archwire Anchorage Force system	MOP protocol	Follow-up/loss	Rate of tooth movement (mm/month)		Outcomes evaluated	Outcome measurements	Conclusions
						Control Maxilla Mean ± SD Mandible Mean ± SD	MOP Maxilla Mean ± SD Mandible Mean ± SD			
Sivarajan et al., Malaysia, 2018 Split-mouth [26]	30, 7/23, 22.2 ± 4.00	Class I, < unit class II, or class III	MBT 0.022 × 0.028″ 0.018 × 0.025″ SS mini-screws; elastomeric chain 140–200 g	Orlus screw (width 1.6 and length 6). Three separate MOPs, vertical direction 2 mm apart and 3 mm in depth. One-time application at the start of retraction	4 m/0	1.02 ± 0.55 NA	1.463 (0.593) -	Clinical measurements using electric digital calipers. Self-administered questionnaire (5-point Likert scale and VAS)	Distance from the central point of the canine bracket to superior margin of the mini-implant (maxilla) and the inferior margin of the mini-implant (mandible); overall pain intensity and impact of any pain on daily function	MOPs were associated with significant increased overall canine retraction over a 16-week period of observation, however not clinically significant; moderate pain was associated with MOP at 4-week intervals; mild pain in 8 and 12 weeks.
Alikhani et al., New York 2013. Split-mouth [12]	20, 8/12, 25.8	Class II div 1	MBT 0.022 × 0.028″ 0.016 × 22″ SS Mini-screws. 100 g NiTi closing coil springs	Propel. Three MOPs distal to the canines, each perforation was 1.5 mm wide and 2 to 3 mm deep. One-time application at the start of retraction	1 m/0	0.45 ± 0.55 NA	1.100 (1.200) -	Cast measurements with digital caliper. Gingival crevicular (GCF) fluids analysis. Rating scale from 1 to 10 for the level of discomfort	The distance between the canine and the lateral incisor in 3 points: incisal, middle, and cervical of the crowns. GCF collected before and after each visit to access the level of inflammatory response. Pain and discomfort levels at 24 h, 7, 14, and 28 days after the beginning of canine retraction	MOPs increased the rate of canine retraction 2–3-fold compared with the control group, reducing treatment time by 62%. Patients reported only mild discomfort locally at the spot of the MOP. At days 14 and 28, little to no pain was experienced.
Attri et al., India, 2018 Parallel control group [25]	60, 27/33, 17.8 ± 2	NA	MBT 0.022 × 0.028″ 0.019 × 0.025″ SS. Trans-palatal arch tie-backs (150 g)	Propel. Three MOPs in the extraction space, 1.5 mm wide and 2–3 mm deep. Repeated after every 28 days until space closure was completed	Until space closure/0	0.58 ± 0.15 0.51 ± 0.10	0.885 (0.180) 0.765 (0.145)	Digital images using a 3D scanning of the plaster models. A 10-mm VAS	The extraction space, a mid-palatine line drawn from distal surface of canine to the mesial surface of the 2nd premolar. Pain perception at 24 h, 7 days, and 28 days after MOP	Acceleration of tooth movement was seen with MOPs. Minimal discomfort was observed post-procedure for participants who had undergone MOPs.
Kundi et al., Saudi Arabian, 2019 Parallel control-group [13]	30, 14/16, 27.9 ± 4.5	Class II div 1	MBT 0.022 × 0.028″ Rectangular wires Banding 1st and 2nd Molars Niti coil springs 100 g	Propel. Three separate MOPs’ distal in the canine, 1.5 mm diameter and 2.5 mm in depth. One-time application at the start of retraction	4 m/0	0.58 ± 0.12 NA	1.492 (0.177) -	Digital images using a 3D scanner of the plaster models. Numerical rating scale (NRS)	The distance between the tip of the canine and the midpoint of incisor edge of lateral incisor, and the cervical midpoints on height of contours of respective cingulum *x* and *y* coordinates on 3D images. Pain intensity at 4 h after the procedure, and after every 24 h for the next 7 days	MOPs accelerated orthodontic tooth movement by 2–3-fold. A significant difference was found in the perception of pain among experimental and control group on the 1st and 2nd days. However, the difference was insignificant during the rest of the week.
Fattori, Brazil, 2020 Parallel control group [19]	18, 7/11, 24.1 ± 6	Severe class III	SLB Roth 0.022″ 019 × 25″ SS Mini-screws 9 mm Niti closed coil spring (200 g)	Propel. Three vertical MOPs in the midway space between canine and 2nd premolar, 6–8 mm deep. Repeated after every activation session until space closure was completed	9 m/control:3 MOP:1	0.61 ± 0.17 NA	0.670 (0.170) -	Digital measurements using Q3DC tool (plaster dental models digitally converted). Treatment time. OHIP-14 questionnaire	Distance from the most distal point in the canine and most mesial point in the 2nd premolar. Closing time between groups (mm/month). Quality of life assessment	Three MOPs were inefficient for accelerating tooth movement during anterior retraction. MOPs produced more impact on OHRQOL immediately following the MOP procedure and after 3 days.
Aboalnaga et al., Egypt, 2019 Split-mouth [15]	18, all females, 20.5 ± 3.9	NA	Roth 0.022″ 0.017 × 0.025 SS wire mini-screws Niti closing coil springs (150 g)	Mini-screw (UNITEK), 1.8 × 8 mm. Three MOPs were performed midway in the extraction space, using a TAD 1.8 × 8mm. One-time application before canine retraction	4 m/0	0.44 ± 0.23 NA	0.532 (0.187) -	Dental models (T0–T4) scanned (images superimpositions). CBCT images before and after treatment. Pain Rating Scale (1–10)	The distance between the canine cuspid tip and frontal plane (FP) in each model (T0–T4). Rate of canine retraction, and the sagittal distance between the mesiobuccal cusp tip of the 1st molar and FP was used to measure anchorage loss. Pain intensity assessed immediately after MOPs, 1 day, 3 days, and 1 week after intervention. Root resorption	MOPs were not able to accelerate the rate of canine; however it seemed to facilitate root movement. Mild to moderate transient pain was experienced following the MOP procedures that almost disappeared 1 week later. No anchorage loss or root resorption was detected.
Haliloglu-Ozkan et al., Turkey, 2018 Parallel control group [18]	32, 19/13, 15.7 ± 1.5	NA	MBT .022 × .028 0.019″ × 0.025″ brass-posted wire mini-screws NiTi closing coil spring 150 g	Mini-screw (MTN-2), 1.6 × 8 mm. Three MOPs were performed in the distal of the canine, depth: 5 mm. Repeated in the 4th week of distalization	2 m/control:3 MOP:1	1.36 ± 0.81 1.01 ± 0.71	1.760 (0.660) 1.020 (0.520)	Plaster models scanned	Canine distalization, canine rotation, and canine tipping and molar mesialization. Anchorage loss	MOPs significantly increased the canine distalization rate at the T1–T3 interval in the maxilla (0.4 mm). The new MOPs performed at T3 did not trigger the canine distalization rate. Tooth movement was faster in the maxilla than in the mandible.
Feizbakhsh et al., Iran, 2018 Split-mouth [27]	20, 12/8, 28	Class I	Roth 0.022 × 0.028 0.019 × 0.025 SS wire 2nd molar. Niti coil 200 g	Mini-screw (Jeil Medical), 1.6 × 3 mm. Two MOPs were performed in 5 mm and 8 mm of crestal bone. Bony screw 1.6 mm diameter and 3 mm length. One-time application at the start of retraction	1 m/0	0.74 ± 0.40 0.53 ± 0.41	1.360 (0.490) 1.240 (0.420)	Plaster models scanned	Distance between the canine and the 2nd premolar measured in three areas: center of the canine and premolar bracket, the canine cusp tip and premolar buccal cusp tip, and the shortest distance between canine and premolar cervico-gingival line	MOPs increased the rate of tooth movement by more than 2-fold when compared to the control side. No significant difference in the rate of tooth movement in the canine retraction, maxilla, and mandible, between interventional and control.
Alkebsi et al., Jordan, 2018 Split-mouth [14]	32, 8/24, 19.3 ± 2.5	Class II div 1	MBT 0.022 × .028 0.019 × 0.025 SS wire Mini-screw NiTi closed coil spring 150 g.	Mini-screw (Aarhus), 1.5 × 6 mm. Three MOPs of 1.5 mm width and 3 to 4 mm, 3 mm distal to canine and 6 mm from the free gingival margin. One-time application before retraction	3 m Contro:1 MOP:1	0.67 ± 0.34 NA	0.650 (0.260) -	3D models obtained every month; clinical measurements using a digital caliper; digital periapical radiographs; VAS; periodontal clinically evaluation	Amount of canine displacement, rate of canine retraction, anchorage loss, canine tipping, canine rotation, root resorption, plaque index, gingival index, pain level, patients’ satisfaction and degree of ease, willingness to repeat the procedure and recommendation to others	Three MOPs were not effective for accelerating tooth movement. No significant differences of anchorage loss, canine rotation, and tipping. Root resorption was similar for both groups. No adverse effect of periodontal health. The level of pain was minimal and faded after 24 h on both sides. MOPs had no effect on the patients’ daily life except for a feeling of swelling on the first day. Patients’ level of satisfaction regarding the MOP was high.
Puetter, Brazil, 2018 Split-mouth [20]	17, 8/9, 16.5 ± 4.4	NA	Edgewise 0.022 × 0.028 0.018 × 0.025 wire, Mini-screws CrNi coil spring 150 g or elastomeric 150 g	Propel. Three MOPs switch a depth of 5 mm distal and parallel to the canine root. One-time application at the start of retraction	2 m/NA	1.26 ± 0.40 NA	1.205 (0.445)	Digital models obtained using a 3D scanner. Panoramic radiographs with radiopaque markers	Anteroposterior distances between canine cusp tips and PMs. Angle formed between the long vertical and mesiodistal axis of the canines. Discomfort questionnaire with four questions, three answered by VAS scale.	MOPs do not accelerate tooth movement significantly. The perception of MOP discomfort was considered mild to moderate.
Alqadasi et al., China, 2019 Split-mouth [28]	8, NA, 15 to 40	Class II div 1	MBT N.A Mini-screw Niti Coil spring 150 g	Automated mini-implant instrumentation. Three perforations of 1.5–2 mm width and 5–7 mm depth in the middle of the extraction space. One-time application at the start of retraction	3 m/NA	0.56 ± 0.41 NA	0.710 (0.400)	Digital images from 3D scanner; photographs and CBCT images; McGill Pain questionnaire	The distance between canine and second premolar; a point on the crown tip and the apex tip; the distance between cementoenamel junction and marginal bone crest from the buccal and lingual sides; numeric scale for pain intensity	MOPs do not significantly speed up tooth movement. Pain and discomfort appear to be exactly similar on both sides. No difference between groups regarding bone height and root resorption.
Babanouri et al., Iran, 2020 Split-mouth [29]	28, 7/5, 26.1 ± 9.1	Class II div 1	MBT 0.022-in 0.016 × 0.022″ SS wire mini-screw NiTi closed coil spring 150 g	Mini-screws 1.2 mm diameter. Three perforations with to a depth of 1 mm, between the distal of the canine and the mesial of the 2nd premolar. The first MOP was located 5 mm away from the free gingival margin. One-time application at the start of retraction	3 m Control: 2 MOP: 2	0.62 ± 0.11	0.76 (0.173)	Plaster models measured using a digital caliper; VAS	The distance between the canine and lateral incisor measured at three points (incisal, middle, and cervical). The amount of pain associated to MOP was evaluated in the day of canine retraction and 24 h later.	MOPs were effective in accelerating tooth movement over a period of 3 months, but not clinically significant. There was no increase in the level of pain and discomfort due to MOPs.

*NA* not available, *MOPs* micro-osteoperforations, *MBT* McLaughlin-Bennet-Trevisi, *SS* stainless steel, *NiTi* niquel-titanium, *OHIP* oral health impact profile, *SD* standard deviation, *GCF* gingival crevicular fluid, *TAD* temporary anchorage device, *CBCT* cone beam computed tomography, *VAS* visual analogue scale

Regarding root resorption, three studies that used conventional mini-screws [14, 15, 28] revealed no differences between the control and experimental groups. Eight studies analyzed the pain associated with the interventions [12–15, 20, 25, 28, 29], only three reported a mild discomfort associated to MOPs after the perforation [13, 15, 20], two with the Propel system [13, 20], and one with other mini-screws [15]; also, five studies, including two with Propel, did not report pain associated to the MOP procedure [12, 14, 25, 28, 29]. The impact on quality of life was evaluated for one study with the Propel system [19] that reported more impact on OHRQoL immediately following the MOP procedure and for 3 days after. Another study [14] evaluated the MOPs impact on daily routine and reported that MOPs had no effect on the patients’ daily life except for a feeling of swelling on the first day. The anchorage loss was evaluated in one study with Propel [13] and in four with conventional mini-screws [14, 15, 18, 28], and did not find differences between the control and experimental groups.

### Synthesis of results

**Fig. 3:**
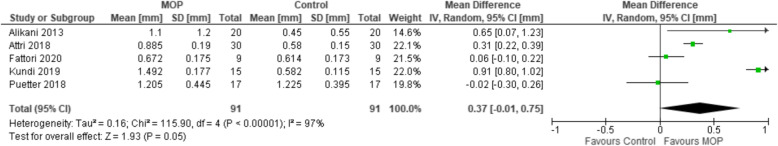
Forest plot for the mean difference of the monthly rate of tooth movement comparing studies that used Propel system

**Fig. 4:**
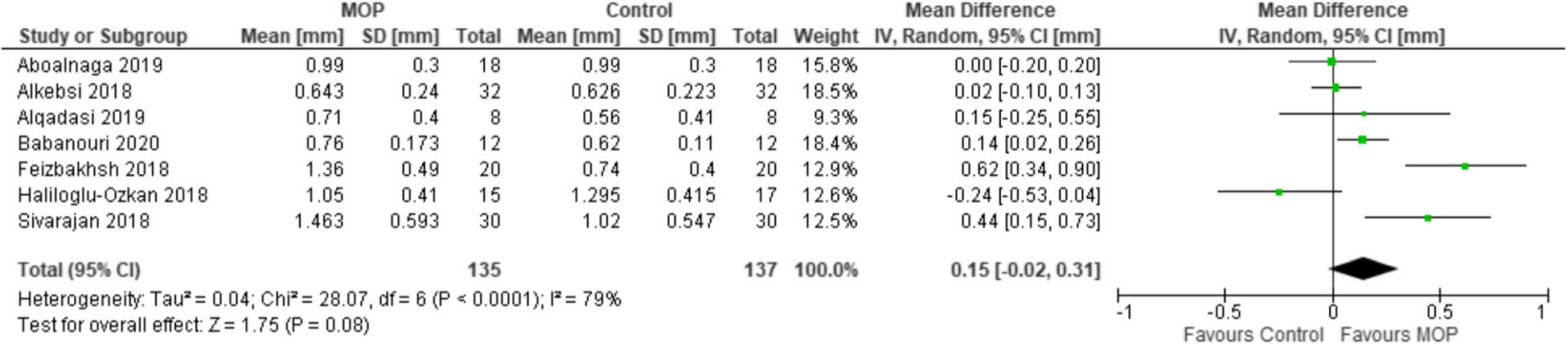
Forest plot for the mean difference of the monthly rate of tooth movement comparing studies that used conventional mini-screws

A random-effect meta-analysis with subgroups was performed using the RevMan 5.3 software; the subgroups were selected according to the perforation system: Propel or conventional mini-screw. The subgroup Propel system included five studies [12, 13, 19, 20, 25] and the another with conventional mini-screws included seven studies [14, 15, 18, 26–29]. Both revealed no statistically significant differences in the monthly rate of tooth movement between the control and MOP groups. The mean difference between the control and MOPs’ group using the Propel system was 0.37 mm (95% CI − 0.01 to 0.75, Fig. 3), and for the group with mini-screws, it was 0.15 mm (95% CI − 0.02 to 0.31, Fig. 4).

### Assessment of the certainty of evidence

The evaluation of the evidence according to GRADE was described in Table 3. The quality of evidence was rated as low for orthodontic tooth movement [12–15, 18–20, 25, 26, 28, 29] and pain or discomfort [12–15, 20, 25, 28] due to inconsistency and imprecision of the studies, as well as the RoB. The outcome related to anchorage loss [13–15, 18] presented a moderate level of certainty, justified by the RoB of the evaluated studies. The GRADE tool rated the outcome root resorption as high [14, 15, 28]. Outcomes investigated by only one study [19] were not evaluated according to GRADE, since this tool aims to analyze the certainty of evidence generated by different studies. Evaluating only one study can lead to an incorrect result. Therefore, quality of life and periodontal outcomes were not analyzed.

**Table 3:** Evaluation of the level of evidence by GRADE PRO assessment tool

Certainty assessment							Impact	Certainty	Importance
No. of studies	Study design	Risk of bias	Inconsistency	Indirectness	Imprecision	Other considerations			
Rate of tooth movement in mm per month									
12	Randomized trials	Serious ^a^	Serious ^b^	Not serious	Not serious	None	Twelve RCTs evaluated the rate of tooth movement. Four showed high RoB, four were classified as some concerns, and four were classified as low RoB. Seven studies related acceleration of tooth movement; however, just two of them showed low risk of bias. Five studies did not find MOPs’ effect on tooth movement.	⨁⨁◯◯ low	Critical
Pain and discomfort									
8	Randomized trials	Serious ^c^	Serious ^d^	Not serious	Not serious	None	Eight RCTs assessed pain or discomfort after MOPs’ procedure. One showed high RoB, three were classified as some concerns, and four presented low RoB. Two RCTs, one showing low RoB and another classified as some concerns, reported pain after the intervention. Six studies did not report pain after the procedure, one with high RoB, two were classified as some concerns, and three with low RoB.	⨁⨁◯◯ low	Critical
Anchorage loss									
5	Randomized trials	Serious ^a^	Not serious	Not serious	Not serious	None	Five RCTs evaluated anchorage loss. One showing high RoB, two were classified as some concerns and two showing low RoB. None of them found differences on anchorage loss between groups.	⨁⨁⨁◯ moderate	Critical
Root resorption									
3	Randomized trials	Not serious	Not serious	Not serious	Not serious	None	Three RCTs assessed root resorption after MOPs’ accomplishment. Two showed low RoB and one was classified as some concerns. None of them found differences on root resorption.	⨁⨁⨁⨁ high	Critical

^a^Haliloglu-Ozkan et al. [18] and Sivarajan et al. [26] presented bias in randomization process; Fattori et al. [19] presented deviations from intended interventions; Puetter et al. [20] showed an error in measurement of the outcome

^b^The studies presented inconsistency in results, some reporting faster orthodontic tooth movement on MOPs’ groups, and others showing no difference between intervention and control

^c^Puetter et al. showed an error in measurement of the outcome

^d^The studies presented inconsistency in the results, some reporting pain after the MOPs’ procedure, others showing no difference between intervention and control

## Discussion

Although three studies with the Propel system have associated the acceleration in rate of tooth movement to MOPs [12, 13, 25], one with low RoB [25] and two with moderate [12, 13], the subgroup meta-analysis did not show statistically significant increases in the monthly rate of tooth movement when MOPs were performed with Propel compared to a control group. Also, the subgroup analysis with mini-screw perforations revealed no differences between the control and MOP groups, as the mean is 0.15 (95% CI − 0.02 to 0.31).

A previous systematic review [16] concluded that the rate of tooth movement increased after performing MOPs in 0.45 mm (95% CI 0.17 to 0.74), in contrast with another review [21] that reported no differences between the mean difference of MOP and control group equal to − 0.01 (95% CI − 0.13 to 0.11). It is important to highlight that a previous review [16] reported an inaccurate sample size [14, 15] and follow-up period [15] of some included studies. Furthermore, this previous review [16] included three studies which used the Propel system [12, 13, 25] and three which used other types of mini-screw [14, 26, 27] in the same meta-analysis.

In this present review, five studies [12, 13, 19, 20, 25] investigated the Propel and seven [14, 15, 18, 26–29] used conventional mini-screws. To see if the use of the Propel system influences the rate of tooth movement, we performed a meta-analysis with subgroups only with studies that evaluated this system. No differences between the control and Propel MOP groups were found. The mean difference was 0.37 mm (95% CI − 0.01 to 0.75, Fig. 3). These findings are not different from those obtained from studies that used other types of mini-screws (mean difference 0.15 mm; 95% CI − 0.02 to 0.31). These results showed that the use of the Propel system, as well as other mini-screws, do not affect orthodontic movement significantly.

Regarding the five studies included in our meta-analysis that used Propel, four [12, 19, 20, 25] measured the outcomes near the date of the perforations. One study [13] performed the MOP once and measured the rate of tooth movement until the space closed, which according to the study seemed to correspond to 4 months and found a higher rate of tooth movement associated to the MOP group. To minimize the effect of time on the quantification of results, we divided the amount of rate of movement in millimeters by the follow-up period. Evaluating the monthly rate of tooth movement during all the follow-up period is done because the acceleration of tooth movement tends to be greater during the first month after the MOP procedure and decreases after that [14, 15].

The evaluated studies have different follow-up periods, orthodontic movement regarding canine retraction and total anterior retraction, frequency of performing MOPs, mechanics used for canine retraction, and measurement methods of tooth movement. Two [12, 27] studies demonstrated accelerated tooth movement in the MOPs’ group and have a shorter investigation time of 28 days. One study [25] followed the movement throughout the total space closure, however expressed an increase in monthly rate of tooth movement of 0.2 mm. The study with the longer follow-up [19] and moderate RoB corresponding to 9 months did not find differences in the monthly rates of tooth movement between the control and MOP groups, even when performing MOPs monthly. Studies with three [14, 28, 29] and four [14] months of follow-up and low RoB reported no acceleration on tooth movement related to MOPs. After the procedure, MOPs might increase the rate of tooth movement, but this effect seems to be clinically insignificant and it is not maintained along the treatment.

Regarding orthodontic movement, ten studies measured the rate of tooth movement associated to canine retraction [12–15, 18, 20, 26–29], which included one with a low and one with a moderate RoB, and using the Propel system evaluated the rate of tooth movement during the anterior retraction [19, 25]. It is important to assess the rate of tooth movement not only during canine retraction, but mainly during anterior retraction, as cases with premolar extraction will require anterior retraction in addition to canine retraction. The data from these two studies [19, 25] report results with greater clinical relevance for determining the choice for performing MOPs during orthodontic treatment. However, one study reported no difference between the control and MOP groups related to the monthly rate of tooth movement [19] and the other reported a mean difference of 0.3 mm between the control and MOP groups. This appears to be a clinically insignificant value to justify the MOPs’ procedure.

Considering the frequency of performing MOPs, one study with a moderate and one with a low RoB performed MOPs with the Propel system once a month [19, 25]. One study with a 4-month follow-up reported acceleration of tooth movement [25]; however, this was clinical insignificant (0.2 mm/month). One study [19] with a 9-month follow-up and a moderate RoB did not find any effects on the monthly rate of tooth movement. Thus, better designed studies that perform MOPs with Propel system every month could be conducted to clarify whether this approach could increase, in a clinically significant way, the rate of tooth movement.

The GRADE analysis classified the certainty of the outcome rate of tooth movement as low due to the presence of bias in the included studies, and due to inconsistency. In fact, the certainty of the evidence would already be compromised no matter how low the RoB was, as several studies have shown divergence in the direction of effect size found. The statistical heterogeneity may reflect the methodological heterogeneity found in the included studies. When comparing the results of this review with others [17, 21], we realize that when a statistically significant effect is found, it has no clinical relevance. Apparently, the system specially developed for performing MOPs, the Propel system, has no impact on the rate of tooth movement.

Great methodological diversity was found concerning the mechanics used for canine retraction. Three RCTs which utilized the Propel system [12, 19, 20] used mini-screws for absolute anchorage and two [13, 25] used conventional anchorage. Of the three studies that used the Propel system and found an increase on the rate of tooth movement, one used mini-screws as anchorage and NiTi springs [12], another used conventional anchorage and NiTi springs [13], and the third used conventional anchorage and tie-backs for canine retraction [25]. These variations in methodologies may have reflected the high heterogeneity found among the results of the included studies.

Complementing the methodological difference, nine studies used scanned models [13–15, 18–20, 25, 27, 28] to perform the measurements, one study evaluated tooth movement through clinical examination [26], and two RCTs took digital caliper measurements on plaster models [12, 29]. Linear measurements made on digital models have similar reliability and accuracy as measurements made on plaster models [30].

The pain reported by patients during the performance of MOPs was investigated by eight studies [12–15, 20, 25, 28, 29]. Three studies with Propel system [12, 20, 25] and two with mini-screw [14, 28] found no difference between pain reported by patients in the control and experimental groups. Three studies with mini-screw perforations [15, 28, 29] and one with Propel system [13] reported pain after performing MOPs, decreasing posteriorly. Pain does not seem to be critically associated to the MOPs’ procedure. Considering the heterogeneity assessment and bias of the studies, the GRADE classified the quality of this evidence as low.

The periodontal condition was evaluated for two studies without the Propel system [14, 28] and both reported no difference between the control and MOP group related to gingival and periodontal index. MOPs had no adverse effect on periodontal health. The assessment of anchorage loss associated with canine retraction was performed by five studies [13–15, 18, 28], which only one used Propel system [13] and no study reported statistically significant differences between control and MOPs’ groups. Two studies with a low RoB [14, 15] and one with a high RoB [18] used skeletal anchorage, which may have influenced the result found. The level of certainty of the anchorage loss was rated by GRADE as moderate. Three studies [14, 15, 28] which performed MOPs with mini-screw, two with low RoB [14, 15], and one with a moderate RoB [28] evaluated the incidence of root resorption and found no significant difference intergroup, confirming the findings in the literature [1, 31]. The level of evidence generated for this outcome is high.

The impact of MOPs on the patients’ daily routine and quality of life was assessed by two studies [14, 19]. One study [19] which used the Propel system and with a moderate RoB showed MOPs produced a greater impact on oral health-related quality of life immediately following the MOP procedure and for 3 days after, affecting mostly “psychological discomfort” and “psychological disability” domains. This variable is influenced by several factors, such as social, economic, and cultural, not addressed by the study, which may lead to an inconclusive outcome. The other study with low a RoB [14] has no statistical difference between the control and the MOP group. These suggest that MOPs have a discrete effect on the patient’s daily routine, limited to the moments after their completion.

## Limitations

The studies sample sizes have great variability which may have influenced the differences between the experimental and MOPs’ groups. Most studies did not follow patients throughout the entire closure of the extraction space; moreover, the procedure was performed only at the beginning of orthodontic retraction in some studies [12–15, 20, 26, 27, 29]. New studies using the Propel system with appropriate sample sizes that evaluate the effects of MOPs throughout the total anterior retraction may generate a higher level of evidence.

Furthermore, it is particularly important to highlight the clinical and methodological heterogeneity observed among the included studies. The differences between the frequencies of MOPs, follow-up periods, perforation systems, and types of retraction reflected a great statistical heterogeneity when performing the meta-analysis. Previously published systematic reviews also present the same limitations [16, 17] or have included only two studies in the meta-analysis [21]. Therefore, it is important to report that a meta-analysis is unfeasible in the presence of high clinical and methodological heterogeneity.

In our systematic review, we tried to control this issue, performing meta-analyses of subgroups, one for the Propel system and one for conventional mini-implants. Furthermore, a monthly average of orthodontic movement due to the different follow-up times was evaluated. After these adjustments, MOPs appear to have no significant clinical effect on the orthodontic movement.

## Conclusion

- Current scientific evidence with low certainty points to no effect of MOPs on orthodontic movement rate when using the PROPEL system, as well as other mini-screws.
- The MOPs seem to have no effect on root resorption, loss of anchorage, periodontal health, and pain/discomfort. They also produced more impact on quality of life immediately following the perforations and for 3 days after.
- A higher level of evidence can be generated with studies which follow the space closure until the end with less methodological heterogeneity.

## Data Availability

The authors declare that all data generated or analyzed during this study are included in this published article and its supplementary information files.

## Appendix

**Table 4:** Search strategy for electronic databases

Database	Keywords	Results
PubMed	((((((((((((((((Orthodontic Tooth Movement[Title/Abstract]) OR Movement, Orthodontic Tooth[Title/Abstract]) OR Movements, Orthodontic Tooth[Title/Abstract]) OR Orthodontic Tooth Movements[Title/Abstract]) OR osteocentesis[Title/Abstract]) OR Tooth Movement, Orthodontic[Title/Abstract]) OR Tooth Movements, Orthodontic[Title/Abstract]) OR tooth movement[Title/Abstract]) OR tooth movements[Title/Abstract]) OR teeth movement[Title/Abstract]) OR teeth movements[Title/Abstract]) OR canine retraction[Title/Abstract]) OR teeth retraction[Title/Abstract]) OR tooth retraction[Title/Abstract])) OR retraction[Title/Abstract])) AND ((((((((Micro-Perforations[Title/Abstract]) OR Micro-perforation[Title/Abstract]) OR Micro-osteoperforations[Title/Abstract]) OR Micro-osteoperforation[Title/Abstract]) OR Flapless osteopuncture[Title/Abstract]) OR Flapless osteopunctures[Title/Abstract]) OR MOPs[Title/Abstract]) OR MOP[Title/Abstract])	29
Scopus	( ( TITLE-ABS-KEY ( "Micro-Perforations" ) OR TITLE-ABS-KEY ( "Micro-perforation" ) OR TITLE-ABS-KEY ( "Micro-osteoperforations" ) OR TITLE-ABS-KEY (osteocentesis) OR TITLE-ABS-KEY ( "Micro-osteoperforation" ) OR TITLE-ABS-KEY ( "Flapless osteopuncture" ) OR TITLE-ABS-KEY ( "Flapless osteopunctures" ) OR TITLE-ABS-KEY ( mops ) OR TITLE-ABS-KEY ( mop ) ) ) AND ( ( TITLE-ABS-KEY ( "Orthodontic Tooth Movement" ) OR TITLE-ABS-KEY ( "Movements, Orthodontic Tooth" ) OR TITLE-ABS-KEY ( "Orthodontic Tooth Movements" ) OR TITLE-ABS-KEY ( "Tooth Movement, Orthodontic" ) OR TITLE-ABS-KEY ( "Tooth Movements, Orthodontic" ) OR TITLE-ABS-KEY ( "tooth movement" ) OR TITLE-ABS-KEY ( "tooth movements" ) OR TITLE-ABS-KEY ( "teeth movement" ) OR TITLE-ABS-KEY ( "teeth movements" ) OR TITLE-ABS-KEY ( "canine retraction" ) OR TITLE-ABS-KEY ( "teeth retraction" ) OR TITLE-ABS-KEY ( "tooth retraction" ) OR TITLE-ABS-KEY ( retraction ) OR TITLE-ABS-KEY ( distalization ) ) )	35
Web of Science	TS = (Orthodontic Tooth Movement OR Movement, Orthodontic Tooth OR Movements, Orthodontic Tooth OR Orthodontic Tooth Movements OR Osteocentesis OR Tooth Movement, Orthodontic OR Tooth Movements, Orthodontic OR tooth movement OR tooth movements OR teeth movement OR teeth movements OR canine retraction OR teeth retraction OR tooth retraction OR retraction) AND TS = (Micro-Perforations OR Micro-perforation OR Micro-osteoperforations OR Micro-osteoperforation OR Flapless osteopuncture OR Flapless osteopunctures OR MOPs OR MOP)	39
Cochrane	#1 Micro-Perforations 3 #2 Micro-perforation 4 #3 Micro-osteoperforations 31 #4 Micro-osteoperforation 28 #5 Flapless osteopuncture 0 #6osteocentesis 0 #7 Flapless 0steopunctures 0 #8 MOPs 73 #9 MOP 249 #10 #1 OR #2 OR #3 OR #4 OR #5 OR #6 OR #7 OR #8 OR #9 334 #11 Orthodontic Tooth Movement623 #12 Movement, Orthodontic Tooth623 #13 Movements, Orthodontic Tooth 79 #14 Orthodontic Tooth Movements 79 #15 Tooth Movement, Orthodontic623 #16Tooth Movements, Orthodontic 79 #17 tooth movement 914 #18 tooth movements221 #19 teeth movement 915 #20 teeth movements 221 #21 canine retraction 146 #22 teeth retraction 231 #23 tooth retraction 231 #24 retraction 1417 #25 distalization 83 #26#11 OR #12 OR #13 OR #14 OR #15 OR #16 OR #17 OR #18 OR #19 #20 OR #21 OR # 22 OR #23 OR #24 OR #25 155470 #27 #10 AND #26 88	88
LILACS	(tw:((tw:(Micro-Perforations)) OR (tw:(Micro-perforation)) OR (tw:(Micro-osteoperforations)) OR (tw:(Micro-osteoperforation)) OR (tw:(Flapless osteopuncture)) OR (tw:(Flapless osteopunctures)) OR (tw:(MOPs)) OR (tw:(osteocentesis)) OR (tw:(MOP)))) AND (tw:((tw:(Orthodontic Tooth Movement)) OR (tw:(Movement, Orthodontic Tooth)) OR (tw:(Movements, Orthodontic Tooth)) OR (tw:(Orthodontic Tooth Movements)) OR (tw:(Tooth Movement, Orthodontic)) OR (tw:(Tooth Movements, Orthodontic)) OR (tw:(tooth movement)) OR (tw:(tooth movements)) OR (tw:(teeth movement)) OR (tw:(teeth movements)) OR (tw:(canine retraction)) OR (tw:(teeth retraction)) OR (tw:(tooth retraction)) OR (tw:(retraction)) OR (tw:(distalization))))	2
Google Scholar	micro-perforations and tooth movement	100
OpenGrey	micro-perforations and tooth movement	0

## Abbreviations

MOPs: Micro-osteoperforations
RoB: Risk of bias
GRADE: Grading of Recommendations Assessment, Development, and Evaluation
RCT: Randomized clinical trial
MBT: McLaughlin-Bennet-Trevisi
NiTi: Niquel-titanium
OHIP: Oral health impact profile
SD: Standard deviation
GCF: Gingival crevicular fluid
TAD: Temporary anchorage device
CBCT: Cone beam computed tomography
VAS: Visual analogue scale
